# Formation of amyloid fibrils by the regulatory 14-3-3*ζ* protein

**DOI:** 10.1098/rsob.230285

**Published:** 2024-01-17

**Authors:** Darius Šulskis, Mantas Žiaunys, Andrius Sakalauskas, Rūta Sniečkutė, Vytautas Smirnovas

**Affiliations:** Institute of Biotechnology, Life Sciences Center, Vilnius University, Vilnius, Lithuania

**Keywords:** amyloid, fibrils, 14-3-3*ζ*, α-synuclein

## Abstract

The 14-3-3 proteins are a highly conserved adaptor protein family with multi-layer functions, abundantly expressed in the brain. The 14-3-3 proteins modulate phosphorylation, regulate enzymatic activity and can act as chaperones. Most importantly, they play an important role in various neurodegenerative disorders due to their vast interaction partners. Particularly, the 14-3-3*ζ* isoform is known to co-localize in aggregation tangles in both Alzheimer's and Parkinson's diseases as a result of protein–protein interactions. These abnormal clumps consist of amyloid fibrils, insoluble aggregates, mainly formed by the amyloid-β, tau and α-synuclein proteins. However, the molecular basis of if and how 14-3-3*ζ* can aggregate into amyloid fibrils is unknown. In this study, we describe the formation of amyloid fibrils by 14-3-3*ζ* using a comprehensive approach that combines bioinformatic tools, amyloid-specific dye binding, secondary structure analysis and atomic force microscopy. The results presented herein characterize the amyloidogenic properties of 14-3-3*ζ* and imply that the well-folded protein undergoes aggregation to β-sheet-rich amyloid fibrils.

## Graphical abstract

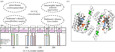

## 1. Introduction

14-3-3 are acidic proteins that first were discovered in bovine brain extracts and titled after their migration position in two-dimensional electrophoresis [1]. Afterwards, these proteins were identified as a highly conserved protein family that is found in all eukaryotes [2]. They have been observed to interact with various kinases and enzymes via recognizing phosphorylation sites [3]. Through the vast interaction network, 14-3-3 proteins regulate the activity and stability of proteins, control localization and facilitate protein–protein interactions (PPIs) [4].

There are seven known mammalian isoforms (*β*, *γ*, *ε*, *η*, *ζ*, *σ* and *τ*/*θ*) of 14-3-3 proteins [5,6], all of which are dimeric. Each monomer is composed of nine anti-parallel α-helices and a disordered C-terminal tail, which is suggested to have an autoinhibitory role against non-specific interactions [7]. Their overall structure resembles a clamp shape with a conserved amphipathic grove, which is used as an accessible binding site for a plethora of proteins [8]. The different interaction specificity of isoforms comes from individual domain movements that ensure flexible adaptations of the binding surfaces [9].

14-3-3 proteins are found in various human tissues [10], despite being expressed most abundantly in the brain tissues [11]. Most prominently, they regulate important physiological functions such as cell survival, differentiation, migration, apoptosis and ion channel regulation [12]. Due to their involvement in numerous crucial roles in the nervous system, they are also associated with neurodegeneration disorders [13]. Different 14-3-3 isoforms have been found in amyloid plaques (neurofibrillary tangles [14] and Lewy bodies [15]), which are indications of ongoing neurodegenerative diseases [16,17]. Although, they seem to have conflicting roles, as one report demonstrates their ability to facilitate the formation of microtubule-associated tau protein fibrils [18], while another study shows their potential to disrupt tau liquid–liquid phase separation and thus inhibit amyloid aggregation [19]. The increased chaperone-like activity of 14-3-3 proteins was observed with Parkinson's disease-related α-synuclein protein [20]. 14-3-3 inhibited α-synuclein aggregation *in vivo* [21] and rerouted its aggregation pathway through binding to intermediate α-synuclein species [22]. Due to these important associations with amyloidogenic and other protein partners, the 14-3-3 family is considered to have a novel therapeutic potential for neurological diseases [16].

While 14-3-3 proteins play a prominent role in neurodegenerative diseases because of their synergy with various partners, it has never been investigated whether they may themselves be susceptible to amyloid aggregation. Previously, there have been several indications that 14-3-3 might be amyloidogenic proteins, since it is known that 14-3-3 share physical and functional homology with α-synuclein [23], particularly in relation to its non-amyloid-β component and C-terminal tail amino acid sequence homology [24]. In addition, it has recently been shown that the concentration of the 14-3-3*ζ* isoform in cerebrospinal fluid increases during the early stages of Alzheimer's disease [25], indicating a potential link with the disorder pathogenesis.

In this study, we investigated whether the 14-3-3*ζ* isoform can aggregate into amyloid fibrils. The preliminary computational data showed that the 14-3-3*ζ* amino acid sequence contains six potential aggregation-prone sites. Subsequently, we monitored protein aggregation using various amyloid-specific dyes and observed gradual, amyloid aggregation over 7 days. The secondary structure analysis confirmed that the exclusively helical protein underwent structural changes and formed β-sheets, akin to the ones found in amyloid fibrils. Finally, atomic force microscopy (AFM) imaging confirmed that 14-3-3*ζ* aggregates consisted of short, curvy, fibril-like structures.

## 2. Results and discussion

### 2.1. 14-3-3*ζ* contains aggregation-prone regions

**Figure 1.**
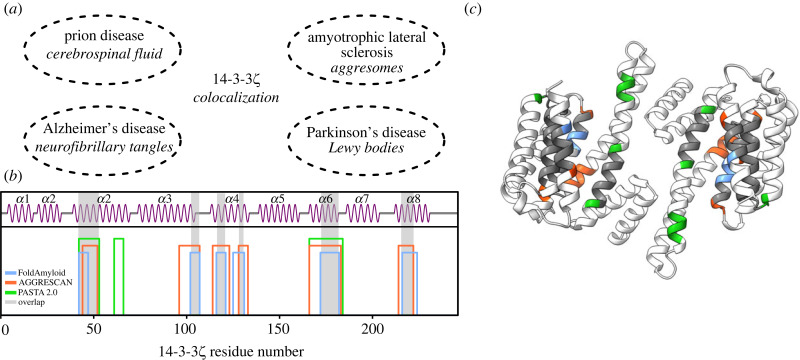
(*a*) 14-3-3*ζ* co-localization sites in various neurogenerative disorders: in cerebrospinal fluid in prion disease [26,27], in aggresomes during amyotrophic lateral sclerosis [28], in neurofibrillary tangles in Alzheimer's disease [14] and in Lewy bodies during Parkinson's disease [29]. (*b*) Predicted aggregation-prone regions by FoldAmyloid (blue), AGGRESCAN (red) and PASTA 2.0 (green). An overlap with at least two predictors is shown with a grey bar. The secondary structure was depicted using Biotite [30]. (*c*) The predicted aggregation-prone sites are located in α-helices of 14-3-3*ζ* (PDB Id: 5NAS). The protein structure was visualized with ChimeraX [31].

14-3-3*ζ* has been located in various plaques of aggregated proteins in different neurodegenerative diseases. It is possible that strong PPI, leads to their co-localization or even co-aggregation with amyloidogenic proteins (figure 1*a*). As 14-3-3*ζ* and other family members share similarities to α-synuclein [23,24], which is a main aggregated component of Lewy bodies [20], they can be suspected to be amyloid-forming proteins. Hence, we analysed the 14-3-3*ζ* amino acid sequence to identify whether 14-3-3*ζ* contains aggregation-prone regions. We used three different predictors of aggregation-prone sites: FoldAmyloid [32], AGGRESCAN [33] and PASTA 2.0 [34]. The aim was to identify whether 14-3-3*ζ* has potential aggregation regions and not to quantify them, therefore, each positive site from at least two predictors was characterized as a hit. Overall, results from AGGRESCAN and FoldAmyloid indicated consistent aggregation-prone regions in six sites, whereas PASTA 2.0 only showed three (figure 1*b*). We identified that the six sites, which overlap between different algorithms, are found in five α-helices of 14-3-3*ζ* (figure 1*b*,*c*). These results matched with previously predicted 14-3-3*σ* aggregation regions in α2, α3 and α8 helices by PASTA 2.0 and AMYLPRED2 [24], while also suggesting the potential involvement of α4 and α6. Since different predictors indicated that 14-3-3*ζ* might aggregate, in order to verify that, we examined 14-3-3*ζ* aggregation properties with various biophysical methods.

### 2.2. 14-3-3*ζ* aggregates bind amyloid-specific dyes

For an initial aggregation control assay, the incubated 14-3-3*ζ* solution was combined with thioflavin-T (ThT) and the sample fluorescence spectra were scanned as described in the Material and methods section. The resulting signal was quite low, which prompted the need for a higher sample concentration prior to the dye assays. The 14-3-3*ζ* aggregates were pelleted and resuspended into a 10 times lower volume before being used in the assays. Furthermore, in order to account for the possibility of non-amyloid fluorescence enhancement of ThT, three additional dye molecules were used, which included 8-anilinonaphthalene-1-sulfonic acid (ANS) (fluorescence increases in hydrophobic environments) [35], Congo red (CR) (absorbance spectrum changes upon binding to amyloid fibrils) [36] and a commercial dye, Amytracker 630, which recently has been shown to have very strong photophysical properties when binding to amyloid fibrils [37].

**Figure 2.**
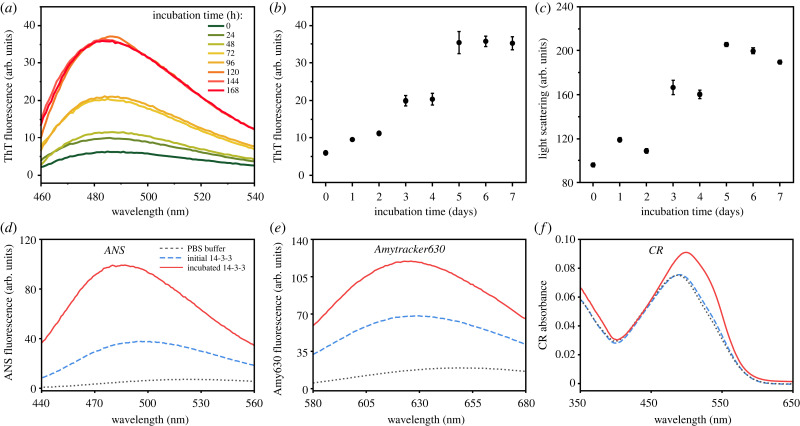
(*a*) The ThT fluorescence spectra of incubated 14-3-3*ζ* solution at different time points. The ThT fluorescence emission (*b*) and light scattering intensity (*c*) over a 7-day period. The ANS (*e*), Amytracker630 (*e*) fluorescence and CR (*f*) absorbance spectra of PBS buffer, initial 14-3-3*ζ* at 0 h and incubated for 168 h 14-3-3*ζ* solutions.

When comparing the intensity of ThT in PBS or with the initial (0 h) 14-3-3*ζ* solution, there were virtually no differences between the spectra (figure 2*a*). Over the 7 days of incubation, we measured the 14-3-3*ζ* solution and observed a significant increase in the fluorescence emission intensity with a maximum at 485 nm (figure 2*a*,*b*). The increase in sample light scattering also confirmed the formation of larger structures (figure 2*c*). In the case of ANS, the aggregated 14-3-3*ζ* solution had the highest signal value (figure 2*d*); however, the 14-3-3*ζ* solution at 0 h also displayed a considerable level of fluorescence. Similar results were obtained when using Amytracker630 (figure 2*e*), suggesting that 14-3-3*ζ* was capable of incorporating these molecules, with most likely them binding in the hydrophobic patch within the binding groove [4]. When CR was combined with the aforementioned solutions, only the incubated 14-3-3*ζ* sample displayed a significant change in the absorbance spectra (figure 2*f*), with the appearance of a shoulder at 540 nm. Such a shift is usually associated with the interaction between CR and amyloid fibrils, as is the increase in total absorbance in the 450–600 nm range [36]. In general, we used four different fluorescence dyes to confirm the amyloid nature of 14-3-3*ζ* aggregates. Both ThT and CR bind cross-β sheet structures in amyloid fibrils [36,38], which indicates that there are structural transitions in the 14-3-3*ζ* protein during aggregation, which could change 14-3-3*ζ* function or interactions with other proteins.

### 2.3. Formation of β-sheets in 14-3-3*ζ* aggregates

**Figure 3.**
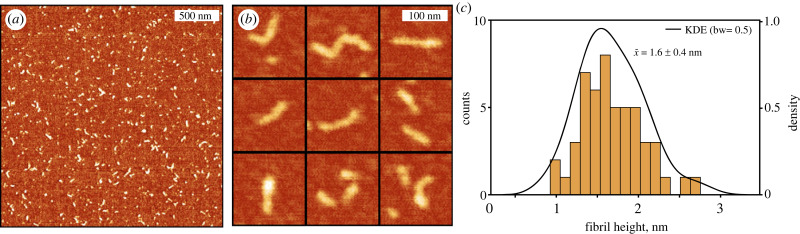
FTIR absorption (*a*), second derivative (*b*) and CD (*c*) spectra of monomeric and aggregate solution of 14-3-3*ζ*.

In the case of monomeric 14-3-3*ζ*, the Fourier-transform infrared spectroscopy (FTIR) spectrum main maximum position was at 1651 cm^−1^ (associated with the presence of α-helical secondary structure [39]) (figure 3*a*). When 14-3-3*ζ* was in its aggregated state, the FTIR spectrum main maximum position shifted towards 1638 cm^−1^ and we observed the appearance of an additional minor band at 1695 cm^−1^ (possibly anti-parallel β-sheets). The second derivative of the monomeric 14-3-3*ζ* FTIR spectrum had a main minimum at 1654 cm^−1^. Oppositely, the main minimum of the aggregated 14-3-3*ζ* FTIR spectrum second derivative was at 1631 cm^−1^ (associated with β-sheets) and two other clear minima at 1657 cm^−1^ (α-helices or turns) and 1695 cm^−1^ (anti-parallel β-sheets) (figure 3*b*).

As complementary to FTIR data, we recorded CD spectra of 14-3-3*ζ* monomers and aggregates (figure 3*c*). The monomer spectrum had two minimum peaks at 209 nm and 223 nm, which is typical for α-helical proteins. On the other hand, the incubated sample spectra had only one unusual red-shifted minimum at 226 nm, which previously had been assigned to β-hairpins in other proteins and peptides [40,41]. This also matches with the observed FTIR band at 1695 cm^−1^, due to the fact that the anti-parallel β-sheet structure is a component of β-hairpins [42].

### 2.4. 14-3-3*ζ* aggregates resemble amyloid fibrils

**Figure 4.**
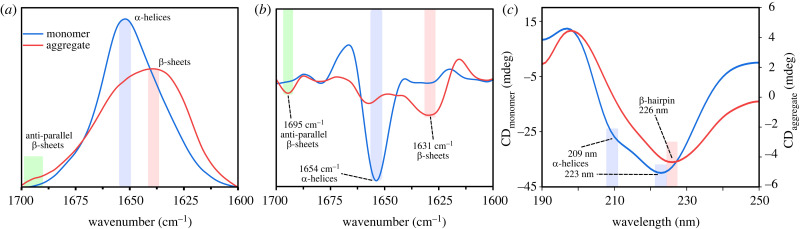
(*a*) AFM image of formed 14-3-3*ζ* amyloid fibrils (scale bar 500 nm). (*b*) Close-up view of fibrils showing curvy morphology. (*c*) Height distribution of fibrils. The black line is the kernel density estimation (KDE) function fit.

As a final confirmation that 14-3-3*ζ* assembled amyloid fibrils, we used AFM imaging to observe the structure and shape of formed aggregates. The initial AFM image showed short amyloid fibrils evenly distributed over the surface of the mica (figure 4*a*). Upon closer inspection, we observed straight or slightly curved fibrils (figure 4*b*). The fibril height distribution was spread between 1 nm and 3 nm, with the mean being 1.6 ± 0.4 nm. During the aggregation assay, we were using α-synuclein fibril formation conditions [43], which included rigorous shaking with glass beads that also could induce fragmentation of aggregates. As an alternative, we tested shaking without glass beads and we observed an increase in ThT fluorescence intensity, but AFM images still only displayed mostly oligomers, with few isolated fibrils (electronic supplementary material, figure S3). Surprisingly, we did find a small number of longer filaments, when it was aggregated at higher protein concentration (200 µM), albeit smaller fibrils still dominated the solution (electronic supplementary material, figure S4). Considering protein concentration might affect protein stability, it may play an important role in determining aggregate length. In all cases, the small shape of the fibrils resembled previously detected worm-like fibrils of the pro-inflammatory S100A9 protein [44] and protofibrils of α-synuclein [45]. These morphological characteristics might be due to large unfolded or still partially folded parts of the protein, which hamper aggregation to long and straight filaments. The FTIR aggregate spectra (figure 3*a*) partially confirmed this, as there was still considerably strong intensity at approximately 1650 cm^−1^, which corresponds to random-coil or α-helices [39].

### 2.5. 14-3-3*ζ* aggregates do not alter α-synuclein aggregation

**Figure 5.**
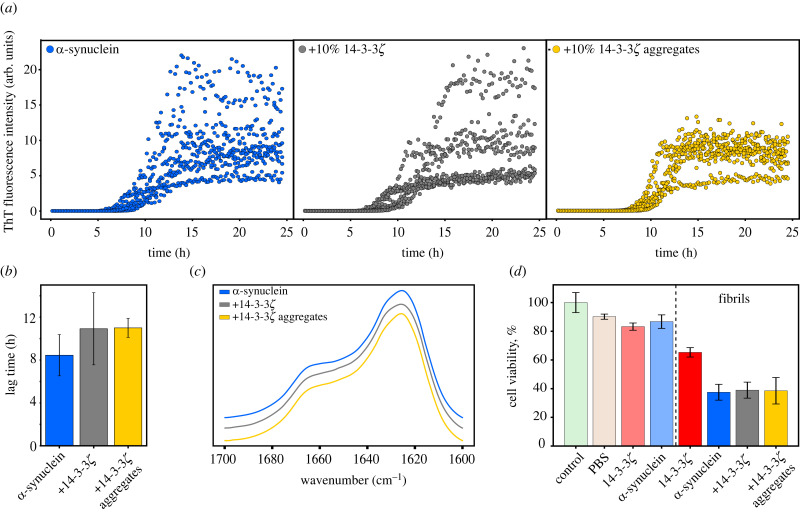
Influence of 14-3-3*ζ* on α-synuclein aggregation. (*a*) ThT fluorescence kinetics, (*b*) calculated lag times and (*c*) FTIR spectra of α-synuclein fibrils, both alone and co-aggregated with 10% 14-3-3*ζ* monomers or aggregates. (*d*) Cell viability MTT tests on SH-SY5Y cell in the presence of 14-3-3*ζ* native protein or aggregates, α-synuclein monomers and α-synuclein fibrils incubated with 10% 14-3-3*ζ* monomers or aggregates.

Since the 14-3-3 protein shares homologous regions with α-synuclein [23], we investigated whether 14-3-3 *ζ* aggregates can impact α-synuclein aggregation. In the ThT fluorescence assay (figure 5*a*) adding 10% of either 14-3-3*ζ* native protein or aggregates increased the mean lag time to 10.9 ± 3.4 h and 11.00 ± 0.9 h, respectively, compared to the control 8.5 ± 1.9 h (figure 5*b*); however, a one-way ANOVA showed no significant differences between different groups (*F* = 0.47, *p* = 0.63, *α* = 0.05). Furthermore, the addition of 14-3-3*ζ* did not change the secondary structure of α-synuclein amyloid fibrils, which indicates that they do not alter the aggregation pathway. AFM imaging also revealed no variations between α-synuclein amyloid fibrils (electronic supplementary material, figure S5). Subsequently, cell toxicity assays of fibrils showed that 14-3-3*ζ* aggregates reduce cell viability to 65% in the MTT assay (figure 5*d*). The α-synuclein with 14-3-3*ζ* monomers or aggregates exhibited the same toxicity as the fibrils alone, consistent with our previous observations that α-synuclein fibrils incubated with 14-3-3*ζ* do not differ from the control.

Overall, we observed that both 14-3-3*ζ* native protein and aggregates had a limited impact on α-synuclein aggregation. These results could be explained by the fact that 14-3-3 isoforms vary in specificity, as previously it has been confirmed that 14-3-3*θ* had much stronger chaperone activity for α-synuclein fibril formation [21]. Another consideration is that the 14-3-3 family interacts with phosphorylated targets [46] and in our experimental conditions α-synuclein was not phosphorylated, making it an incompatible target. Nevertheless, the more important part is that 14-3-3*ζ* aggregates alone can be toxic to the cells and this warrants further experiments to elucidate 14-3-3*ζ* potential aggregation inside the cell.

## 3. Conclusion

In this study, for the first time, we observed the formation of amyloid fibrils by 14-3-3*ζ*. The small shape morphology and relatively weak fluorescence of bound amyloidogenic dyes indicate that 14-3-3*ζ* fibrils are not easily detectable but exhibit properties that are found in amyloids. Additionally, they were toxic to neuroblastoma cells, but could not accelerate a α-synuclein aggregation, although they share amino acid sequence similarity [24]. Since the detection of 14-3-3 proteins is often associated with the onset of neurodegenerative diseases [25,26,47], our study suggests that this might not only be due to their neuroprotection[48] and chaperone [49] roles but also due to the formation of 14-3-3 fibrils. Moreover, with recent studies indicating 14-3-3 involvement in liquid–liquid phase separation, it is becoming evident that 14-3-3 might have an even larger role in neurodegenerative disorders than initially thought [50,51]. As it is now, the revelation of 14-3-3*ζ* amyloid properties opens up new possibilities for further investigations into other isoforms of 14-3-3 and their relationship to other protein aggregation pathways.

## 4. Material and methods

### 4.1. Protein expression and purification

The 6xHis-SUMO-14-3-3*ζ* construct used in this work (kind gift of Prof. B.M. Burmann) was derived from GST-14-3-3*ζ* (Addgene no. 13278) [46]. The plasmid containing 6xHis-SUMO-14-3-3*ζ* was chemically transformed into One Shot *Escherichia coli* BL21 Star (DE3) (Fisher Scientific) cells. The transformed cells were grown at 37°C in LB medium containing kanamycin (50 mg ml^−1^) until an optical density at 600 nm ≈ 0.7 was reached. Expression was induced by the addition of 0.4 mM isopropyl-β-d-thiogalactopyranoside (Fisher Scientific), and the cells were left to grow overnight at 25°C. Cells were harvested by centrifugation at 6000*g* for 20 min at 4°C and subsequently resuspended in 50 ml of lysis buffer (25 mM Hepes/NaOH, 1 M NaCl and 10 mM imidazole (pH 7.5)). The suspension was lysed with a Sonopuls (Bandelin) homogenizer (10 s on, 30 s off, 30% power, total time 30 min). Cell debris was removed by centrifugation at 18 000*g* for 45 min at 4°C, and the supernatant was applied to a Ni^2+^ Sepharose 6 Fast Flow (GE Healthcare) loaded gravity column, followed by stepwise elution with 20 ml of lysis buffer supplemented with 100 and 500 mM imidazole, respectively. Fractions containing the 6xHis-SUMO-14-3-3*ζ* protein were dialysed against phosphate-buffered saline (PBS, pH 7.4), and the 6xHis-SUMO tag was removed by enzymatic cleavage using human sentrin-specific protease 1 (SENP1) catalytic domain (derived from pET28a-HsSENP1) that was a gift from Jorge Eduardo Azevedo (Addgene plasmid no. 71465) at 4°C overnight [52,53]. The primers used for the isolation of the SENP1 catalytic domain gene can be found in electronic supplementary material, table S1. The cleaved proteins were applied again to an Ni^2+^ column, and the flow-through was collected. The proteins were concentrated using Amicon centrifugal filters (10k molecular weight cut-off (MWCO), Merck Millipore) and purified further by size exclusion chromatography (Superdex 75, GE Healthcare) in PBS. The α-synuclein was purified as described previously [54], concentrated to 600 µM and stored at −20°C.

### 4.2. Aggregation-prone sequence analysis

Predicted 14-3-3*ζ* aggregation-prone regions were calculated using three different predictors: PASTA 2.0 [34], FoldAmyloid [32] and AGGRESCAN [33]. The default parameters were used for each prediction. Briefly, in PASTA 2.0 90% sensitivity and −2.8 energy cut-off were used. In FoldAmyloid, aggregation sites were positive, if five successive amino acids with a score above 21.4 were detected. AGGRESCAN identified hot spots of aggregation, whenever the window of five amino acids had an amino acid aggregation-propensity value higher than −0.02. The raw data of predictors results are presented in electronic supplementary material, figure S1.

### 4.3. 14-3-3*ζ* and α-synuclein aggregation

The purified 14-3-3*ζ* or α-synuclein was diluted to 100 µM/200 µM or 100 µM, respectively, using a PBS pH 7.4 solution and distributed to 2.0 ml non-binding test tubes (400 µl volume, each test tube contained two 3 mm glass beads) or Corning non-binding 96-well plates (Fisher, Waltham, MA, USA, cat. no. 10438082) (80 µl volume, each well containing none or one 3 mm glass bead). The samples in tubes were then incubated at 37°C with constant orbital 600 r.p.m. agitation. Every 24 h, three samples were taken for analysis. Samples from 14-3-3*ζ* 200 µM were taken after 3 and 72 h for AFM imaging. The plates were placed in a ClarioStar Plus plate reader (BMG Labtech, Ortenberg, Germany). The ThT fluorescence measurements were taken every 10 min using 440 nm excitation and 480 nm emission wavelengths every 10 min with constant orbital shaking (600 r.p.m.) in-between at 37°C.

The α-synuclein stock solutions were thawed at room temperature and combined with PBS, 10 mM ThT and monomeric or aggregated 14-3-3 samples (100 µM) to a final α-synuclein concentration of 100 µM, 100 µM ThT and 10 µM 14-3-3. Control solutions contained PBS in place of 14-3-3. Samples were placed in 96-well non-binding plates (100 µl solution, each well contained one 3 mm glass bead). For each condition, six repeats were measured in a ClarioStar Plus plate reader as described before.

### 4.4. Fluorescence and absorbance assays

For dye-binding assays, ThT, ANS and CR powders were dissolved in PBS buffer solutions and filtered using 0.22 µm syringe filters. The final concentrations of the dye solutions were set to 20 µM (ThT − *ε*_412_ = 23 250 M^−1^ cm^−1^, ANS – *ε*_351_ = 5 900 M^−1^ cm^−1^, CR – *ε*_486_ = 33 300 M^−1^ cm^−1^) based on their specific absorbance spectra, which were scanned using a Shimadzu UV-1800 spectrophotometer. Amytracker630 stock solution was diluted 40 times using PBS buffer prior to use. The prepared dye solutions were stored at 4°C in the dark.

The dye solutions were combined with either PBS, the 14-3-3*ζ* solution at 0 h or 168 h in a 1 : 1 ratio. The mixtures were then incubated for 10 min in the dark. The fluorescence spectra of ThT, ANS and Amytracker630 were scanned using a Varian Cary Eclipse spectrofluorometer with 10 nm excitation and emission slits, 1 s averaging time and 1 nm intervals (ThT—440 nm excitation and 460–540 emission range, ANS—370 nm excitation and 420–560 emission range, Amytracker630—480 nm excitation and 580–680 nm emission range). The absorbance of CR was scanned from 200 nm to 800 nm using a Shimadzu UV-1800 spectrophotometer. All spectra were corrected using control samples, which did not contain the dye molecules.

### 4.5. Light scattering assay

Sample right-angle light scattering was scanned with a Varian Cary Eclipse spectrofluorometer, using 600 nm excitation and emission wavelengths with 2.5 nm slit widths, 1 s averaging time.

### 4.6. Fourier-transform infrared spectroscopy experiments

The aggregated 14-3-3*ζ* samples (2 ml volume, 100 µM initial protein concentration) and α-synuclein samples (combined six 100 µM repeats of 100 µl initial protein concentration), were centrifuged for 15 min at 12 000*g*. Afterwards, the supernatant was carefully removed and replaced with 500 µl D_2_O with 400 mM NaCl (the addition of NaCl improves aggregate sedimentation). The centrifugation and resuspension procedure was repeated three times. After the final centrifugation, the aggregate pellet was resuspended into 50 µl of D_2_O with NaCl. The suspension was then scanned as described previously [55] using a Bruker Invenio S FTIR spectrometer. Data analysis was carried out with GRAMS software. D_2_O and water vapour spectra were subtracted from the sample spectrum, which was then normalized between 1700 cm^−1^ and 1600 cm^−1^.

To acquire the FTIR spectra of monomeric 14-3-3*ζ*, the buffer solution (PBS, pH 7.4) was exchanged into D_2_O with 400 mM NaCl using a 10 kDa protein concentrator. The protein solution was diluted to 100 µM using the D_2_O solution, placed in the concentrator (400 µl volume) and centrifuged for 10 min at 9000 r.p.m. The concentrated protein solution (approx. 50 µl) was then diluted to the original volume with the addition of 350 µl D_2_O. This centrifugation and dilution procedure was repeated four times. The final concentrate was diluted to 100 µl and used for FTIR analysis. The spectra were obtained and analysed the same as the 14-3-3*ζ* aggregate sample.

### 4.7. Circular dichroism spectroscopy

All measurements were performed on a Jasco J-815 spectrometer at room temperature. Briefly, either a freshly prepared monomeric solution of 100 µM 14-3-3*ζ* (PBS, pH 7.4) or aggregated sample was transferred to a 0.1 mm quartz cuvette. Spectra were measured at 1 nm data pitch from 190 nm to 250 nm with a bandwidth of 2 nm and scanning speed of 50 nm min^−1^. The final spectra of each sample were averaged from three scans with the buffer background subtracted. Analysis and visualization of spectra were done in Spectragryph software (http://spectragryph.com).

### 4.8. Atomic force microscopy measurements

AFM imaging was done similarly to that previously described [56]. In brief, a 40 µl sample of aggregated 14-3-3*ζ* was placed on freshly cleaved mica that was functionalized using 40 µl of APTES ((3-Aminopropyl) triethoxysilane), incubated for 5 min. Then the mica was washed with 2 ml MiliQ water and dried gently under a stream of air. High-resolution images (1024 × 1024) were collected using Dimension Icon (Bruker) AFM operating in tapping mode (Tap300AI-G silicon cantilever (40 N^−1^ m^−1^, Budget Sensors)). AFM image flattening and fibril analysis were done using Gwyddion v2.57 [57]. The additional AFM images of 14-3-3*ζ* aggregates can be found in electronic supplementary material, figure S2.

### 4.9. Cell culturing

SH-SY5Y human neuroblastoma cells were obtained from the American Type Culture Collection (ATCC, Manassas, VA, USA). The cells were grown in Dulbecco's modified Eagle's medium (DMEM) (Gibco, Grand Island, NY, USA), supplemented with 10% fetal bovine serum (FBS) (Sigma-Aldrich, St Louis, MO, USA), 1% penicillin–streptomycin (10 000 U ml^−1^) (Gibco) at 37°C in a humidified, 5% CO_2_ atmosphere in a CO_2_ incubator.

### 4.10. Cytotoxicity of 14-3-3 fibrils

For the MTT assay, SH-SY5Y cells were seeded in a 96-well plate (approx. 15 000 cells well^−1^) and cultured overnight. The 14-3-3*ζ*, α-synuclein monomers or fibrils in PBS were diluted to a final concentration of 5 µM with DMEM and used to replace the cell medium in each well. After 48 h of incubation, 10 µM of MTT was added to each well and left to incubate for 2 h. One hundred microlitres of 10% SDS with 0.01 M HCl solution was added on top to dissolve formazan crystals. Absorbances at 540 nm, 570 nm and 690 nm (reference wavelength) of each well were measured using a ClarioStar Plus plate reader (BMG Labtech).

## Data Availability

The kinetic and FTIR, CD data used for analysis have been tabulated and are available on Mendeley Data: 10.17632/564277pjyx.1. All other relevant data are available from the corresponding author upon reasonable request. Supplementary material is available online [58].
